# Formulation Changes Affect Material Properties and Cell Behavior in HA-Based Hydrogels

**DOI:** 10.1155/2012/737421

**Published:** 2012-11-27

**Authors:** Thomas Lawyer, Kristen McIntosh, Cristian Clavijo, Lydia Potekhina, Brenda K. Mann

**Affiliations:** ^1^Department of Bioengineering, University of Utah, 36 S. Wasatch Drive, Rm. 3100, Salt Lake City, UT 84112, USA; ^2^SentrX Animal Care, Inc., 615 Arapeen Drive, Suite 110, Salt Lake City, UT 84108, USA

## Abstract

To develop and optimize new scaffold materials for tissue engineering applications, it is important to understand how changes to the scaffold affect the cells that will interact with that scaffold. In this study, we used a hyaluronic acid- (HA-) based hydrogel as a synthetic extracellular matrix, containing modified HA (CMHA-S), modified gelatin (Gtn-S), and a crosslinker (PEGda). By varying the concentrations of these components, we were able to change the gelation time, enzymatic degradation, and compressive modulus of the hydrogel. These changes also affected fibroblast spreading within the hydrogels and differentially affected the proliferation and metabolic activity of fibroblasts and mesenchymal stem cells (MSCs). In particular, PEGda concentration had the greatest influence on gelation time, compressive modulus, and cell spreading. MSCs appeared to require a longer period of adjustment to the new microenvironment of the hydrogels than fibroblasts. Fibroblasts were able to proliferate in all formulations over the course of two weeks, but MSCs did not. Metabolic activity changed for each cell type during the two weeks depending on the formulation. These results highlight the importance of determining the effect of matrix composition changes on a particular cell type of interest in order to optimize the formulation for a given application.

## 1. Introduction

Tissue engineering continues to grow as a field, and with it the number of potential scaffolds also grows. Among the plethora of synthetic and natural scaffolds available are synthetic extracellular matrices (ECMs), scaffolds that are generally a mixture of multiple components and are meant to mimic various functions of the natural ECM. These functions include both structural support and cellular signaling, thereby influencing cell shape, fate, and metabolism. Thus, the goal of optimizing a synthetic ECM should be to direct cell function toward recapitulating a natural ECM, and therefore a natural tissue.

Hyaluronic acid (HA) is one component that has been used in synthetic ECMs due to the benefits that HA provides to the body, including water homeostasis, joint lubrication, and its role during development and wound healing processes [1–7]. Crosslinked hydrogels based on HA remain a promising tool for a wide range of applications [8]. For example, HA-based hydrogels have been used for skin and corneal wound healing, postsurgical adhesion prevention, and as scaffolds for tissue engineering and in vitro 3D cell culture applications [9–13]. 

There are many methods available for crosslinking HA to create hydrogel scaffolds, including crosslinking with divinyl sulfone or 1,4-butanediol diglycidyl ether, and photocrosslinking of (meth)acrylated HA [14–17]. However, the method we have focused on covalently attaches thiol groups to the HA and then crosslinks it with either disulfide bonds directly linking one HA to another or using a crosslinking molecule containing thiol-reactive groups, such as poly(ethylene glycol) diacrylate (PEGda). For tissue engineering scaffolds, thiolated gelatin has often been added to this mix in order to allow cell attachment [9, 18–21], although cell adhesion peptides or protein fragments can also be used [22, 23]. The system has the flexibility to modify both the physical and (bio)chemical properties of the scaffold by altering the type and degree of crosslinking, the molecular weight of the HA and crosslinker, and the concentration of each component.

Previous studies were sometimes based on a single formulation, despite being used for different applications such as bone or osteochondral repair and tumor engineering [19–21]. Although such a simplistic approach would make manufacturing easier, it is not likely that a one-size-fits-all formulation is best for different cells and/or tissues. To this point, the effect of changing the composition of these hydrogels on the overall hydrogel properties and on cells seeded within the hydrogels has not been fully studied. Although one study examined the rheological properties only of HA-based hydrogels, the range of formulations used did not cover those in the previously mentioned articles [24].

Here we have varied the composition of these HA-based hydrogels to create a family of formulations and examined the effect on gelation time, enzymatic degradation, and compressive modulus. We also seeded fibroblasts and mesenchymal stem cells within the hydrogels to determine the effect of changing composition on cell spreading, cell number, and metabolic activity. We hope to use this information, coupled with information from future studies on intracellular signaling for a given cell type, in order to more systematically optimize these HA-based hydrogels for specific applications.

## 2. Materials and Methods

### 2.1. CMHA-S Synthesis

Medical device-grade HA (900 kDa; Novozymes Biopolymers, Inc., Bagsvaerd, Denmark) was dissolved in 45% NaOH and stirred at room temperature for 2.5 hours. This mixture was then placed in isopropanol, and chloroacetic acid dissolved in isopropanol was added and allowed to react for 1 hour, then settle out of solution for 30 minutes. The liquid was decanted, and the resultant carboxymethyl HA (CMHA) was dissolved in deionized (DI) H_2_O. The pH was adjusted to 7.0, and the CMHA was purified using tangential flow filtration (TFF).

3,3′-Dithiobis(propanoic dihydrazide) (DTP; Arké Organics, Fornacette, Italy) was added to the purified CMHA solution and the pH adjusted to 4.75. *N*-Ethyl-*N* ^'^-(3-dimethylaminopropyl)carbodiimide (EDAC; Sigma-Aldrich, St. Louis, MO) was then added and the pH maintained at 4.75 until a gel had formed, which was allowed to react for a total of 4 hours. Dithiothreitol (DTT; Gold Biotechnology, St. Louis, MO) was added, the pH adjusted to 8.5 and stirred overnight. The resultant thiolated CMHA (CMHA-S) was purified with TFF. CMHA-S was then lyophilized and stored at −80°C. MW (305 kDa) was assessed using GPC and dynamic light scattering. The level of thiol modification (7.5 × 10^−4^ ± 0.5 × 10^−4^ mmol thiol/mg CMHA-S) was assessed using 5,5′-dithio-bis(2-nitrobenzoic acid) (Ellman's reagent; Sigma-Aldrich).

### 2.2. Gtn-S Synthesis

Endotoxin-free porcine-derived type A gelatin (250 Bloom, Gelita, Sioux City, IA) was dissolved in DI H_2_O and thiolated using the same protocol as for thiolating CMHA described above using DTP, EDAC, and DTT. The resultant thiolated gelatin (Gtn-S) was also purified using TFF, lyophilized, and stored at −80°C. Thiol modification was assessed using Ellman's reagent and determined to be 3.0 × 10^−4^ ± 0.1 × 10^−4^ mmol thiol/mg Gtn-S.

### 2.3. PEGda Synthesis

PEG (MW 3350; Sigma-Aldrich) was acrylated as previously described [25], except that the resultant PEGda was purified by dialysis. Acrylation was verified using ^1^H-NMR [26].

### 2.4. Hydrogel Formation and Gelation Time

Six hydrogel formulations were created, with the final concentrations of components as given in Table 1. To create the hydrogels, the CMHA-S and Gtn-S were dissolved together in phosphate-buffered saline (PBS, pH 7.4). PEGda was dissolved separately in PBS. Once dissolved, the PEGda solution was then added to the CMHA-S/Gtn-S solution and mixed gently by inversion. When cells were seeded within the hydrogels, each solution was filter sterilized using a 0.2 *μ*m filter prior to use. For gelation time determination, a gel volume of 1 mL was placed in a 2 mL microcentrifuge tube. The tube was capped and inverted once every minute to mix and check for gelation. The gelation time was determined as the time at which the mixture would no longer flow with gravity [26, 27]. Six replicates were used for each formulation.

### 2.5. Enzymatic Degradation

Hydrogels were prepared as above, except the CMHA-S/Gtn-S/PEGda solution was placed in a 5 cm × 5 cm × 2 mm silicone mold and allowed to gel for 1 hr at 37°C in a humidified environment. Disks (8 mm diameter) were punched out of the hydrogel and placed in a 24-well plate. Hydrogels were placed in PBS for 24 h at 37°C to swell to their equilibrium state. The PBS was then replaced with fresh PBS or PBS containing 5, 10, or 50 U/mL hyaluronidase (bovine testicular, Sigma-Aldrich); 0.2, 0.5, or 2.0 mg/mL collagenase (bacterial, Sigma-Aldrich); or a combination of 5 U/mL hyaluronidase and 0.2 mg/mL collagenase. Six replicates of each hydrogel formulation with each enzyme concentration were used. The samples were incubated at 37°C for the duration of the experiment, until each hydrogel had fully degraded or until 814 hrs when the experiment was stopped. At each timepoint, the enzyme solution was removed, the hydrogel was blotted to remove excess liquid and weighed, and fresh enzyme solution was added. Timepoints were: every 10 minutes up to the first hour; 3, 5, 8, and 24 hrs; every 24 hrs after that up to 408 hrs; every 72 hrs after that. Hydrogels were deemed to be fully degraded when a single complete piece could no longer be detected in or removed from the solution.

### 2.6. Compressive Modulus

Hydrogels were prepared as for enzymatic degradation, except they were prepared in 35 mm diameter polystyrene Petri dishes. Hydrogels were tested within 2 hours after the 1 hr gelation period. Compressive modulus was determined using an Instron 3342 (Instron, Norwood, MA) under confined compression. A custom-made spindle was used that fit the inner diameter of the Petri dish and could be fixed to the upper clamp of the testing apparatus. Materials were compressed with a crosshead speed of 200 *μ*m/min. Five samples of each formulation were tested. Compressive modulus was determined from the linear portion of the stress-strain curve.

### 2.7. Cell Seeding

Human dermal fibroblasts (HDFs; Lonza, Walkersville, MD) were maintained on low-glucose Dulbecco's modified Eagle medium supplemented with 10% fetal bovine serum, 2 mM L-glutamine, 500 U penicillin, and 100 mg/L streptomycin (DMEM complete). Human mesenchymal stem cells (MSCs; Lonza) were maintained on Mesenchymal Stem Cell Growth Medium (MSCGM; Lonza). Cells were incubated at 37°C/5% CO_2_ and passaged weekly. HDFs were used for experiments at passages 5-10; MSCs were used at passages 3–5. Cells were seeded within hydrogels by resuspending a cell pellet in the filter-sterilized CMHA-S/Gtn-S solution. Filter-sterilized PEGda solution was then added, and the resultant cell-polymer suspension was aliquoted into a 96-well plate (50 *μ*L/well). The final cell seeding density in the hydrogels was 1.5 × 10^6^ cells/mL for HDFs, except as indicated below, and 0.5 × 10^6^ cells/mL for MSCs. Following gelation, DMEM complete or MSCGM was placed on top of the gels as appropriate for the cell type and changed every 2-3 days.

### 2.8. Cell Analysis

Pictures were taken of HDFs in the hydrogels at days 1 and 7 to qualitatively assess differences in cell spreading among the formulations. For both cell types, DNA within the gels was determined using a CyQuant NF assay kit (Invitrogen, Carlsbad, CA), and metabolic activity was determined using a CellTiter96 AQueous One Solution kit (Promega, Madison, WI). Six replicates for each hydrogel formulation for each assay (except the MTS assay with MSCs which had eight replicates) at each timepoint (1, 4, 7 or 8, and 14 (HDFs) or 15 (MSCs) days after seeding) were analyzed. Gels without cells were used as background controls. 

Cell numbers within the hydrogels are compared herein according to the relative fluorescence in the CyQuant assay. Metabolic activity per cell is expressed herein as the relative absorbance from the MTS assay divided by the average relative fluorescence from the CyQuant assay.

In order to determine whether cell seeding density influences metabolic activity within the hydrogels, the experiment was repeated with HDFs using a single formulation (F in Table 1) and seeding densities of 0.4 × 10^6^, 0.8 × 10^6^, 1.5 × 10^6^, and 3.0 × 10^6^ cells/mL.

### 2.9. Statistical Analysis

Unpaired, two-tailed *t*-tests were used to determine statistical significance, *P* ≤ 0.05, between formulations and seeding densities. Comparisons for cell number and metabolic activity were made between the different formulations at a particular timepoint. For the cell density study, comparisons were made between the seeding densities at each timepoint and for each seeding density between timepoints. All values reported are mean ± standard deviation.

## 3. Results and Discussion

### 3.1. Gelation Time

**Figure 1:**
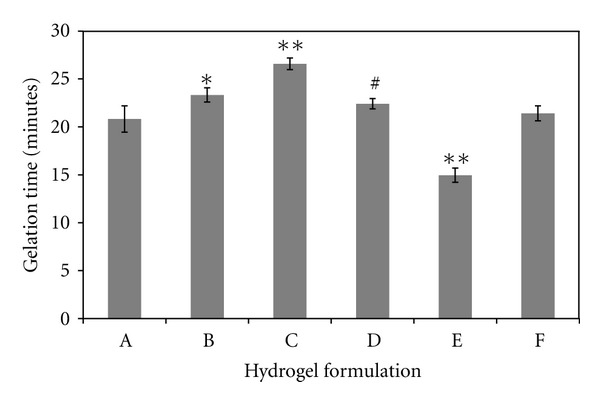
Time required for each formulation to form a gel, as determined when the solution would no longer flow under the force of gravity. *N* = 6 for each formulation; values are mean ± SD. *P < 0.01 compared to A and F; ^#^ *P* < 0.05 compared to A, B, and F; **P ≪ 0.01 compared to all other formulations.

The gelation times of the six hydrogel formulations tested here ranged from 15 minutes to 26 minutes (see Figure 1). Four of the formulations (A, B, D, and F) had similar gelation times, while E was significantly faster and C significantly slower than the others. One of the primary factors influencing gelation time was the concentration of PEGda, demonstrated by the significantly faster crosslinking of formulation E compared to the others. This was to be expected due to the increase in the concentration of acrylate groups available for reacting with the thiol groups. Interestingly, C gelled significantly slower than A or B, despite having the same thiol : acryl ratio. This may be due to differences in the concentrations of CMHA-S versus Gtn-S among those three formulations, coupled with the differences in MW of CMHA-S compared to Gtn-S. The concentrations of the components in Table 1 are provided on a weight basis, yet the MW of CMHA-S is 6x higher than that of Gtn-S. Thus, on a molar basis, the concentrations of CMHA-S and Gtn-S in C are 0.04 and 0.16 *μ*mol/mL, respectively, while they are 0.03 and 0.24 *μ*mol/mL in A. Despite keeping the thiol : acryl ratios the same, the differences in molar concentrations of the biopolymers may lead to differences in accessibility of the thiols to the acrylate groups, thereby affecting the gelation time.

### 3.2. Enzymatic Degradation

**Figure 2:**
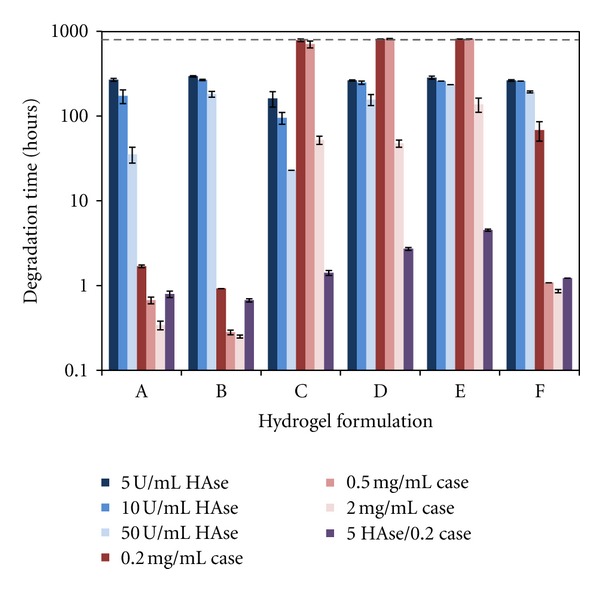
Time required to fully degrade hydrogels using 5, 10, or 50 U/mL hyaluronidase (HAse) in PBS; 0.2, 0.5, or 2.0 mg/mL collagenase (Case) in PBS; or a combination of 5 U/mL HAse and 0.2 mg/mL Case in PBS. Hydrogels in PBS without enzyme did not degrade. The dashed line indicates the point at which the experiment was terminated (814 hrs). *N* = 6 for each formulation in each enzyme solution; values are mean ± SD.

All six hydrogel formulations were able to degrade in the presence of hyaluronidase and/or collagenase (Figure 2). They followed a typical course of hydrogel degradation [28] in which the weight of the gel increased in the initial phases as more water entered the gel, then decreased until the gel finally degraded completely (data not shown). Over the course of this study, none of these formulations displayed significant hydrolytic degradation (i.e., no enzyme present). Two of the formulations, D and E, did not completely degrade by the end of the study (814 hrs) with the two lowest concentrations of collagenase.

As expected, an increase in enzyme concentration led to a decrease in the time required for complete degradation of the hydrogels. In the presence of hyaluronidase, degradation time increased as PEGda concentration increased due to increased crosslinking within the gel, while degradation time decreased with increasing CMHA-S concentration. Thus, E (highest PEGda) degraded slowest and C (highest CMHA-S) degraded fastest in hyaluronidase.

In the presence of collagenase, again the degradation time increased as PEGda concentration increased, and degradation time decreased as Gtn-S concentration increased. Thus, E (highest PEGda) degraded slowest and B (highest Gtn-S coupled with lowest PEGda) degraded fastest in collagenase.

With both enzymes present, even though they were at their lowest concentrations, a synergistic effect led to significantly faster degradation for all of the formulations. This effect was particularly pronounced for gels C, D, E, and F.

### 3.3. Compressive Modulus

**Figure 3:**
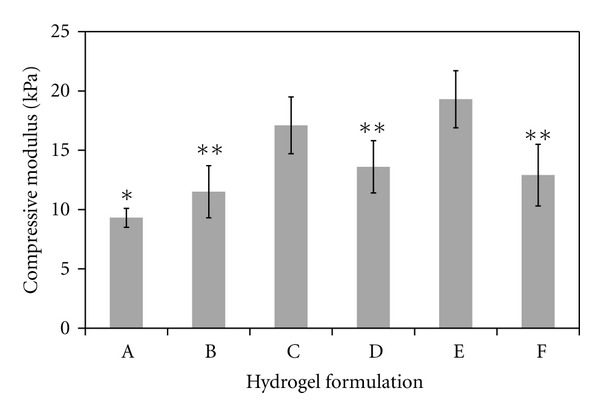
Compressive modulus of each hydrogel formulation. *N* = 5 for each formulation; values are mean ± SD. *P < 0.05 compared to C, D, E, and F; **P < 0.01 compared to C and E.

The compressive modulus of each formulation is given in Figure 3. Formulation C, with a higher concentration of CMHA-S and lower concentration of Gtn-S, had a significantly higher compressive modulus compared to A or B. Although the amount of crosslinking should be the same due to having the same thiol : acryl ratio, these results indicate that the properties of individual components can affect the overall modulus. Similar results were found in a previous study, where an increasing amount of Gtn-S, without changing the amount of CMHA-S, did not change the overall shear modulus of the hydrogel [24]. In other words, the presence or absence of the gelatin has less influence on the overall properties of the hydrogel than does the presence of CMHA-S. In this case, by replacing some CMHA-S with more Gtn-S, the compressive modulus decreases. Additionally, in this study an increase in PEGda (comparing formulation A to D and E) led to an increase in compressive modulus, which is likely due to a combination of both increased crosslinking (lower thiol : acryl ratio) as well as the presence of more PEGda.

### 3.4. Cell Spreading

**Figure 4:**
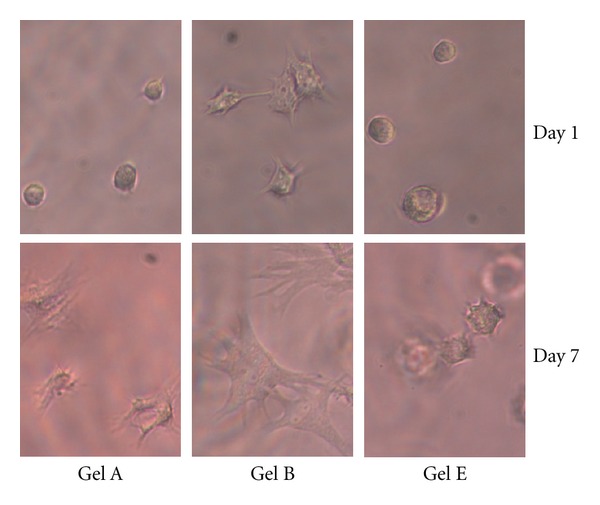
Qualitative representation of spreading of HDFs seeded within three of the hydrogel formulations (A, B, and E) for 1 and 7 days. Formulation A is shown as the base formulation, B is shown as it allowed the most cell spreading, and E is shown as it allowed the least cell spreading.

Fibroblasts seeded within the hydrogels were able to spread. This spreading increased over time, and the degree to which they spread was dependent on the formulation.  Figure 4 shows images of cells at 1 and 7 days, in formulations A, B, and E. These formulations represent the initial formulation, the one with the highest Gtn-S concentration, and the one with the highest PEGda concentration, respectively, and illustrate the extremes of low and high degrees of spreading within the hydrogels.

With an increasing concentration of Gtn-S in the hydrogel, fibroblasts spread more quickly and to a greater degree, most likely due to the presence of more cell adhesion sites. On the other hand, as the concentration of PEGda in the hydrogel increased, fibroblast spreading decreased. With the increased amount of crosslinking in the gel, a tighter network is formed and may make it more difficult for the cells to extend processes and access the cell adhesion sites. Further, formulation E has a significantly greater compressive modulus than A or B, and this physical characteristic of the gel may also influence the cell spreading, as has been shown for cells seeded on top of hydrogels [25, 29].

### 3.5. HDF Proliferation and Metabolic Activity

**Figure 5:**
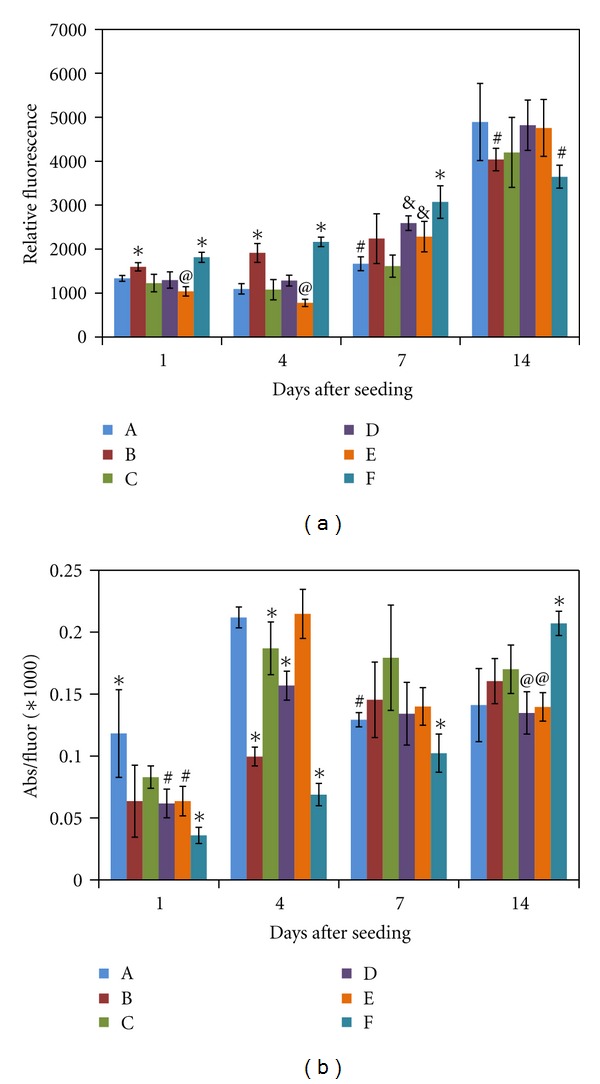
(a) Number of HDFs within the hydrogel formulations as determined using the CyQuant assay, and provided as relative fluorescence. *P < 0.05 compared to all other formulations; ^@^ *P* < 0.05 compared to A and D; ^#^ *P* < 0.05 compared to all except C; ^&^ *P* < 0.05 compared to C. (b) Metabolic activity per cell for HDFs seeded within the hydrogel formulations. Metabolic activity was determined using an MTS assay, providing relative absorbance, which was then divided by the average relative fluorescence from the CyQuant assay. *P < 0.05 compared to all other formulations; ^@^ *P* < 0.05 compared to B and C; ^#^ *P* < 0.05 compared to C. *N* = 6 for each formulation and each assay at each timepoint; values are mean ± SD. Statistical analysis is within each timepoint.

In order to assess how changes in the formulation may affect the function of cells seeded within them, we determined both cell number (relative fluorescence in a CyQuant assay) and metabolic activity per cell (relative absorbance in an MTS assay divided by the relative fluorescence). Although seeded with the same number of cells in each formulation, there is already variability in cell number by day 1 (see Figure 5(a)). Cell numbers in the various formulations, in general, increase over the course of 2 weeks. However, there are some significant differences among the formulations. Notably, there is a significant drop in cell number between day 1 and day 4 in formulation E. Recall that this is the formulation with the highest PEGda concentration, resulting in the lowest thiol : acryl ratio, and thereby the highest degree of crosslinking and highest compressive modulus. It is also the formulation in which the cells were least spread. Initially, through day 4, cell numbers are significantly higher in gels B and F, both of which have high concentrations of Gtn-S. By day 14, cell numbers in the other formulations have equaled or surpassed those in B and F. These results suggest that crosslinking density, access to cell adhesion sites, and compressive modulus may all affect the ability of the cells to proliferate within a three-dimensional hydrogel.

The metabolic activity of the fibroblasts also changes significantly when placed into the different hydrogel formulations. As shown in Figure 5(b), the activity per cell varies among the formulations at all-time points. Activity per cell is highest in Gel A at day 1, but highest in Gel F at day 14. In Gels B and F, the metabolic activity per cell steadily increases over the 14 days. In other formulations, the activity per cell either increases significantly from days 1 to 4, then decreases from 4 to 7 and levels off (Gels A, D, and E), or simply increases from days 1 to 4 then levels off. These results suggest that the Gtn-S may have the biggest influence over how metabolically active the cells in the hydrogel are. 

**Figure 6:**
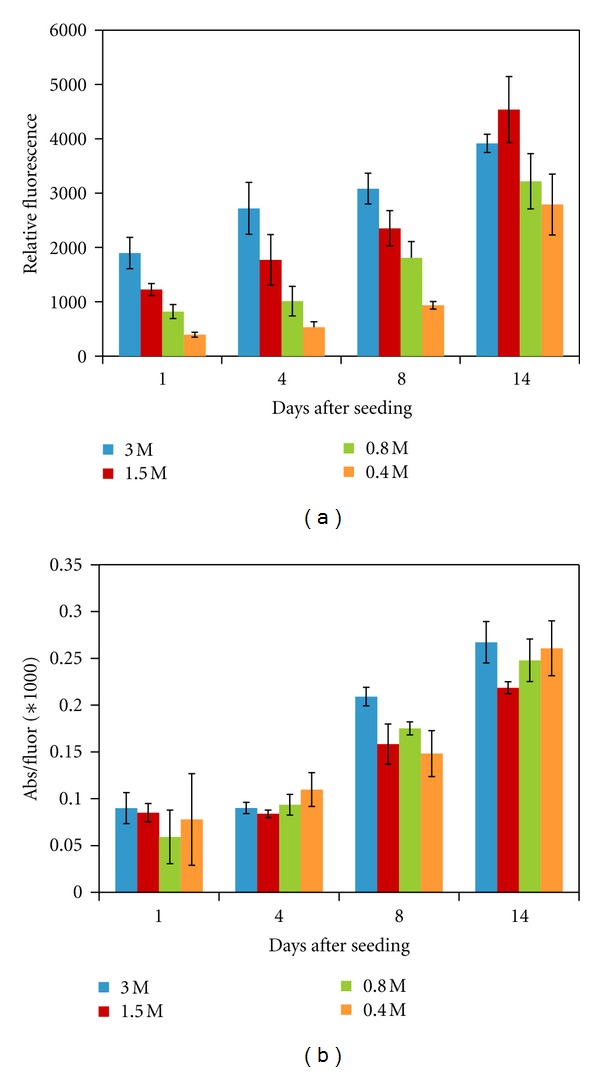
(a) Number of HDFs within formulation F as determined using the CyQuant assay, and provided as relative fluorescence, using initial cell seeding densities of 0.4, 0.8, 1.5, and 3.0 million cells/mL. (b) Metabolic activity per cell for HDFs seeded within formulation F using the different cell seeding densities. Metabolic activity was determined using an MTS assay, providing relative absorbance, which was then divided by the average relative fluorescence from the CyQuant assay. *P < 0.05 compared to all other formulations. *N* = 6 for each seeding density and each assay at each timepoint; values are mean ± SD. Statistical analysis is within each timepoint.

We next wanted to determine whether initial seeding density would influence the metabolic activity of the cells. For this, we chose formulation F and seeded fibroblasts at four seeding densities: 0.4, 0.8, 1.5, and 3.0 × 10^6^ cells/mL. As shown in Figure 6(a), the number of cells within the gels increases in the expected manner based on seeding density. Cell number continues to increase over the course of 14 days with all four seeding densities. However, at day 14, the number of cells in gels seeded with the two highest seeding densities are not significantly different. This may suggest that there is a maximum number of cells that the gel will support at this time. The metabolic activity per cell is very similar among all seeding densities at all time points (see Figure 6(b)), indicating that the seeding density does not affect the metabolic activity of the cells.

### 3.6. MSC Proliferation and Metabolic Activity

**Figure 7:**
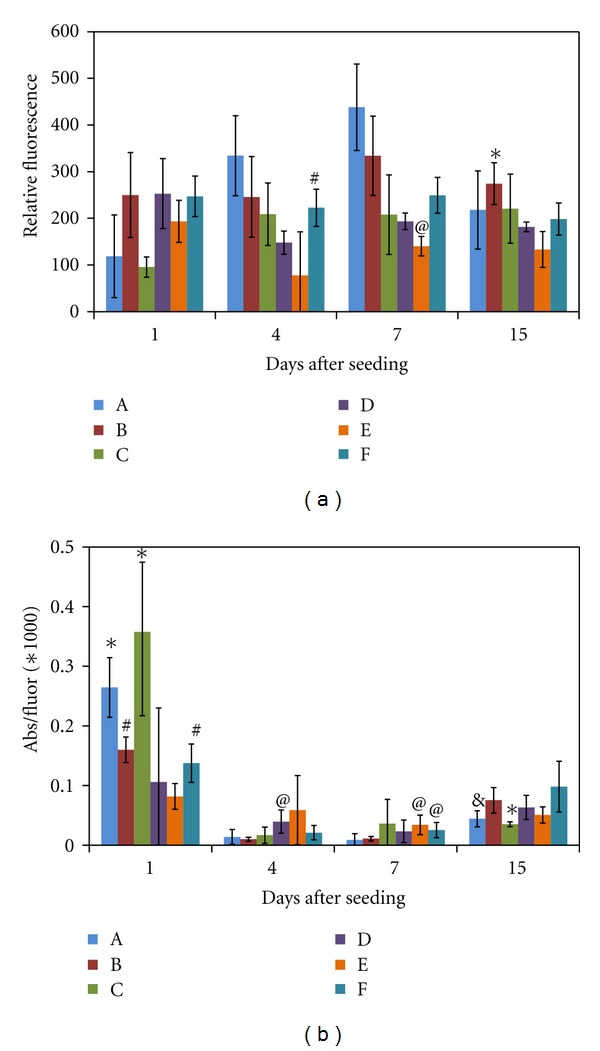
(a) Number of MSCs within the hydrogel formulations as determined using the CyQuant assay and provided as relative fluorescence. *P < 0.05 compared to all other formulations except A and C; ^#^ *P* < 0.05 compared to D; ^@^ *P* < 0.05 compared to all other formulations. (b) Metabolic activity per cell for MSCs seeded within the hydrogel formulations. Metabolic activity was determined using an MTS assay, providing relative absorbance, which was then divided by the average relative fluorescence from the CyQuant assay. *P < 0.05 compared to all formulations except A and C; ^#^ *P* < 0.05 compared to E; ^@^ *P* < 0.05 compared to A and B; ^&^ *P* < 0.05 compared to B and F. *N* = 8 for the CyQuant assay and *N* = 6 for the MTS assay for each formulation at each timepoint; values are mean ± SD. Statistical analysis is within each timepoint.

We also wanted to investigate the influence of the formulation on a different cell type. We chose mesenchymal stem cells for this as they are increasingly being studied for use in tissue engineering and regenerative medicine applications. As seen in Figure 7(a), cell number once again depends on the gel formulation. However, unlike the HDFs, cell number does not consistently increase over the course of the 2-week experiment. In fact, formulation C, with the lowest Gtn-S and highest CMHA-S, is the only one in which the cell number is significantly higher at day 14 than at day 1. It is possible that the MSCs need more time to recover being switched from a 2D to a 3D environment or any new microenvironment. It is also possible that these hydrogel formulations are not optimal for supporting proliferation of MSCs, and, for example, a much softer material or one that provides other signaling may be more suitable. There was also a lot more variability in the cell number for the MSCs than the HDFs, as reflected in the larger standard deviations. One possible explanation is that the MSCs were not distributed as evenly in the cell-polymer mixture prior to aliquoting, leading to replicates with different cell densities initially. Such uneven distribution could be due to the much lower seeding density used for the MSCs compared to the HDFs, or potentially due to stronger cell-cell adhesion between MSCs, making them more difficult to separate and therefore distribute in the mixture.

The metabolic activity per cell for MSCs also varied significantly between formulations (see Figure 7(b)). At day 1, the highest activity per cell was found in gels A and C, which had the lowest cell numbers. Unlike the HDFs, the metabolic activity per cell dropped significantly from day 1 to day 4 for MSCs in all formulations except D and E, in which the activity per cell decreased, but was not significant due to high variability between replicates. This low activity per cell persisted through day 7, then increased somewhat by day 14. These results may indicate that the MSCs require a different period of adjustment to their new microenvironment than do the HDFs, at least in the case of these hydrogels.

When the cells, whether HDFs or MSCs, were in standard tissue culture for maintenance and expansion, the cells were on a very stiff material—polystyrene—and had attachments to the surface on only one side of the cell (2D). The integrins ligated in this case are likely dominated by *α*_5_ *β*_1_, connected to RGD on fibronectin [30, 31] adsorbed to the surface from serum in the medium [32]. These cells were then moved to a much softer material—the hydrogel—where they can form attachments on many sides (3D), and the integrins ligated are likely *α*_*v*_ *β*_3_, connected to an RGD on gelatin [33, 34]. Although in both environments attachments are made to an RGD site, different integrins are primarily used with the different proteins due to different surrounding amino acids or synergistic sites [35]. The change from a stiff to a soft material, the change from 2D to 3D, and the change in cell adhesion could each cause the cells to undergo an adaptation phase in which they must adjust to their new microenvironment, resulting in intracellular signaling changes [36]. Although other cells have been found to utilize *α*_*v*_ *β*_3_ in order to attach to gelatin [37, 38] (which is different than attachment to collagen I [33, 34]), it is not as clear for MSCs. One group did not find *α*_*v*_ *β*_3_ expressed by MSCs [39] while another group found it expressed, but only by 20% of cells [40]. If it is indeed expressed only at low levels, this could account for the drop in cell number by MSCs in the first week within the hydrogels. It is possible that the cells remaining make a recovery of sorts by expressing their own fibronectin, as embryonic stem cells can [41]. Previous studies have shown that MSCs require ligation of *α*_5_ *β*_1_ for certain cell functions [40, 42], and loss of this when moving them from the 2D cell culture to these 3D hydrogels may lead to the proliferation and metabolic activity changes observed here.

From these results, the formulation that may be best for proliferation might not be best for metabolic activity; the formulation that appears best at day 1 might not be best at day 14. This could be due, in part, to remodeling aspects. HDFs and MSCs are likely to produce different amounts of MMPs and other enzymes that can degrade the gel, thus leading to differential abilities to remodel their surroundings. Further, the system is more complex than it initially may seem. For example, the presence of Gtn-S and being covalently crosslinked into the network affect spatial issues and movement of molecules through the gel, compressive modulus, and cell adhesion. Following ligation to an adhesive sequence, the integrin used to attach to the gelatin then begins a signaling cascade through the cell. However, the cell can also sense the stiffness of the material upon such ligation, which in turn influences cell behavior. For example, it has recently been shown that integrin-specific interaction with the ECM coupled with ECM mechanics work synergistically to direct behavior of lung epithelial cells [43]. Additionally, chondrocytes have been shown to alter MMP and ECM production in PEG-based hydrogels, depending on the degree of crosslinking [44]. Thus, if we change the concentration of Gtn-S, a change in cell behavior could be due to the change in cell adhesion sites, stiffness of the material, ability to access nutrients, or some combination of these. Although we may not fully understand all of the interactions, if we know that increased Gtn-S leads to early survival of a particular cell type, yet a stiffer hydrogel would be more beneficial later, then we may be able to make modifications to the formulation, or even to the Gtn-S itself, to achieve both aims. Further studies investigating both enzyme production by the cells for remodeling the hydrogel, as well as production of new ECM proteins will be important for ultimately determining the optimal formulation for a particular cell type and with a specific application in mind.

## 4. Conclusions

Tissue engineering and regenerative medicine application often rely on the use of a biomaterial in conjunction with cells. This biomaterial may serve multiple functions, including structural support and signaling. Thus, the design of the biomaterial in directing cell function to achieve a desired result is important. Synthetic extracellular matrices can serve as the biomaterial in these applications, and one that we have focused on combines a modified hyaluronic acid (CMHA-S) with a modified ECM protein (Gtn-S) and a synthetic polymer (PEGda), covalently crosslinked to form a hydrogel. By varying the three components, we were able to change physical aspects of the hydrogel, such as gelation time and compressive modulus, and biochemical aspects, such as enzymatic degradation rate and cell adhesion sites. These variations led to changes in cell proliferation and metabolic activity, which was also dependent on cell type. Combined with future studies on remodeling of the synthetic ECM and recapitulation of new tissue, these results may be useful in further development of this family of biomaterials for specific cells or tissues.

## Figures and Tables

**Table 1:** Composition of the hydrogel formulations used in this study. Formulation A is the same as that used in [19].

Formulation	PEGda (mg/mL)	CMHA-S (mg/mL)	Gtn-S (mg/mL)	Thiol : Acrylate
A	8	10	12	2.83 : 1
B	8	7	16	2.83 : 1
C	8	13	8	2.83 : 1
D	12	10	12	1.89 : 1
E	16	10	12	1.42 : 1
F	12	7	16	1.89 : 1
